# Supplementary material for: Enhanced Electromagnetic Wave Absorption of SiOC/Porous Carbon Composites
Source: Materials (Basel). 2022 Dec 12;15(24):8864. doi: 10.3390/ma15248864 (PMC9782895; doi:10.3390/ma15248864)
Supplement: Supplementary file 1 [file materials-15-08864-s001.zip › materials-2027994-supplementary.pdf]

Supporting Information

Enhanced Electromagnetic Wave Absorption of SiOC/Porous Carbon Composites

Wen Yang [1,†], Li Li [2,†], Yongzhao Hou [3,], Yun Liu [4,*] and Xinwei Xiao [3]*

1 School of Transportation and Vehicle Engineering, Shandong University of Technology, Zibo 255000, China

2 Shandong Si-nano Materials Technology Co., Ltd., Zibo 255400, China

3 School of Materials Science and Engineering, Shandong University of Technology, Zibo 255000, China

4 Shandong Industrial Ceramics Research & Design Institute Co., Ltd., Zibo 255400, China

† Equal contribution

** correspondence: houyz1990@sdut.edu.cn (Y.H.); LY17864301063@163.com (Y.L.); Tel.: +86-18816307735 (Y.H.)*

Table S1. Main properties of the raw pitch.

Sample	C%	H%	N%	S%	SP(℃)	QI%	TI%	Ash%
Pitch	92.97	4.595	0.92	0.515	87.6℃	0.4%	13.8	0.038

Note: SP: softening point; QI: quinolone insoluble; TI: toluene insoluble

Table S2. Band wavenumbers and assignments in the FTIR spectra.

Position/cm^{-1}	Assignments
	Pitch
3042	aromatic C-H stretching vibration
2980-2800	C-H stretching vibration
1594	C=C stretching absorption
1500-1300	C-H bending vibrations absorption
700-900	C-H stretching and out-of-plane bending vibrations
	PSA
2171	-Si-H stretching vibrational
2042	C≡C stretching vibrational
1259	Si-CH_3 vibrations.

Table S3. EDS results of Porous C/sp-SiOC-8.

Spectrum 1			Spectrum 2			Spectrum 3		
element	Wt (%)	At (%)	element	Wt (%)	At (%)	element	Wt (%)	At (%)
Si	14.70	7.10	Si	14.56	7.03	Si	26.86	13.82
C	73.12	82.57	C	73.18	82.58	C	67.05	80.68
O	12.18	10.33	O	12.26	10.39	O	6.08	5.50

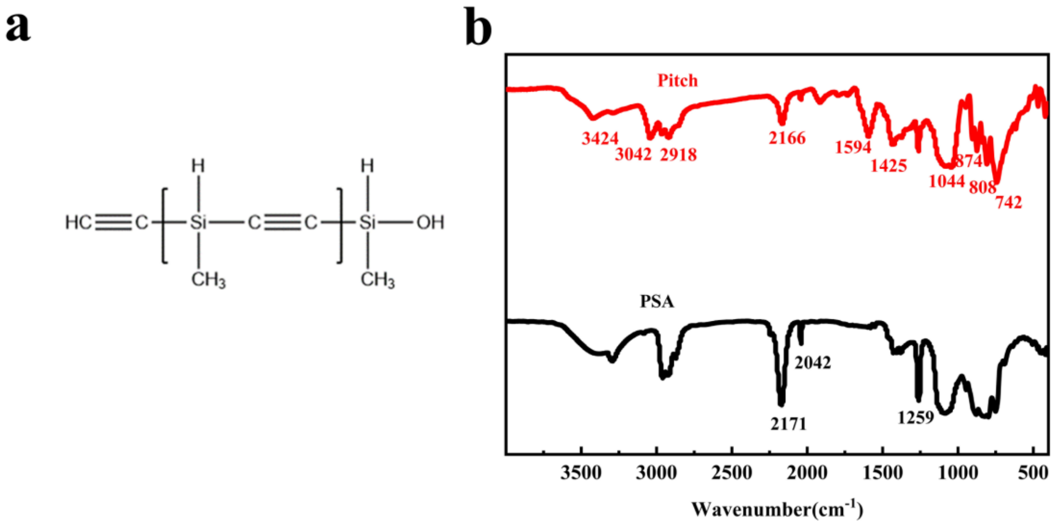

Figure S1. (a) Schematic diagram of the molecular structure of polysilyacelene. (b) FTIR spectra of raw materials(Pitch; PSA) and composites(Pitch/PSA).

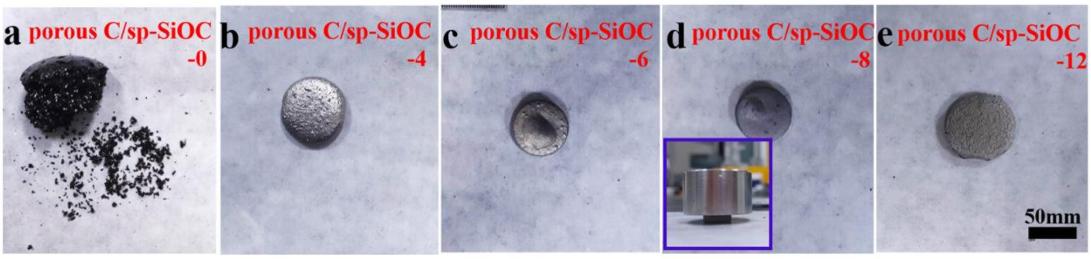

Figure S2. Photo of (a) Porous C/sp-SiOC-0, (b) Porous C/sp-SiOC-4, (c) Porous C/sp-SiOC-6, (d) Porous C/sp-SiOC-8, (e) Porous C/sp-SiOC-12.

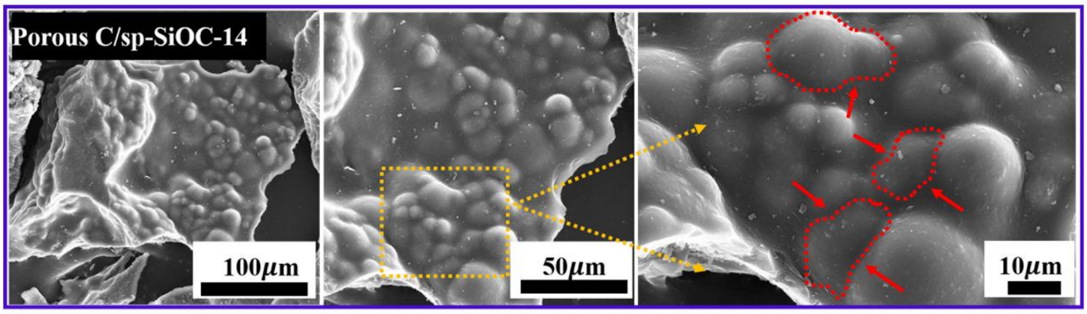

Figure S3. SEM images of carbonized products of Porous C/sp-SiOC-14 composite at 1000℃.

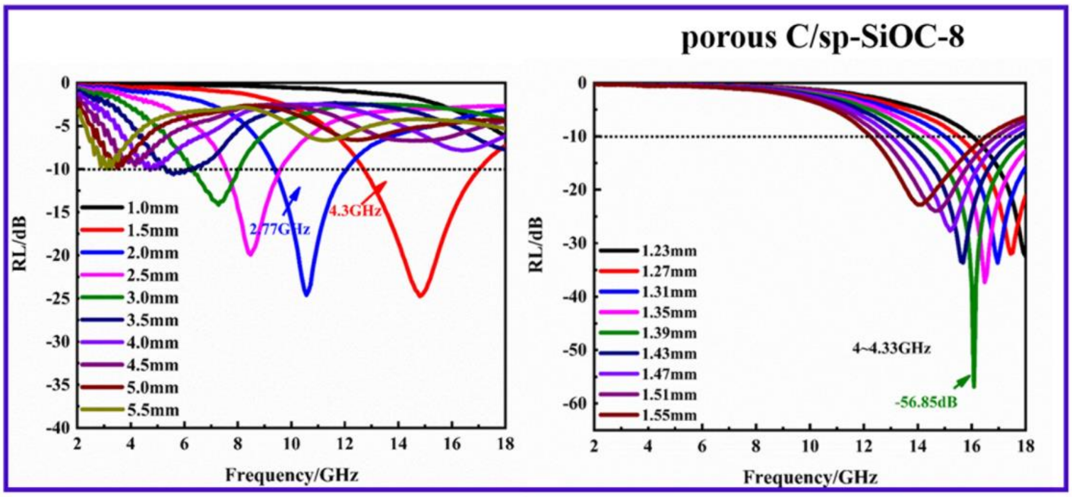

Figure S4. Minimum RL of the Porous C/sp-SiOC-8,
